# Modeling Intracerebral Hemorrhage Growth and Response to Anticoagulation

**DOI:** 10.1371/journal.pone.0048458

**Published:** 2012-10-30

**Authors:** Charles H. Greenberg, Matthew P. Frosch, Joshua N. Goldstein, Jonathan Rosand, Steven M. Greenberg

**Affiliations:** 1 Department of Bioengineering and Therapeutic Sciences, Department of Pharmaceutical Chemistry, and California Institute for Quantitative Biosciences, University of California San Francisco, San Francisco, California, United States of America; 2 C. S. Kubik Laboratory in Neuropathology, Massachusetts General Hospital, Harvard Medical School, Boston, Massachusetts, United States of America; 3 Department of Emergency Medicine, Massachusetts General Hospital, Harvard Medical School, Boston, Massachusetts, United States of America; 4 Department of Neurology, Massachusetts General Hospital, Harvard Medical School, Boston, Massachusetts, United States of America; University of Regensburg, Germany

## Abstract

The mechanism for hemorrhage enlargement in the brain, a key determinant of patient outcome following hemorrhagic stroke, is unknown. We performed computer-based stochastic simulation of one proposed mechanism, in which hemorrhages grow in “domino” fashion via secondary shearing of neighboring vessel segments. Hemorrhages were simulated by creating an initial site of primary bleeding and an associated risk of secondary rupture at adjacent sites that decayed over time. Under particular combinations of parameters for likelihood of secondary rupture and time-dependent decay, a subset of lesions expanded, creating a bimodal distribution of microbleeds and macrobleeds. Systematic variation of the model to simulate anticoagulation yielded increases in both macrobleed occurrence (26.9%, 53.2%, and 70.0% of all hemorrhagic events under conditions simulating no, low-level, and high-level anticoagulation) and final hemorrhage size (median volumes 111, 276, and 412 under the same three conditions), consistent with data from patients with anticoagulant-related brain hemorrhages. Reversal from simulated high-level anticoagulation to normal coagulation was able to reduce final hemorrhage size only if applied relatively early in the course of hemorrhage expansion. These findings suggest that a model based on a secondary shearing mechanism can account for some of the clinically observed properties of intracerebral hemorrhage, including the bimodal distribution of volumes and the enhanced hemorrhage growth seen with anticoagulation. Future iterations of this model may be useful for elucidating the effects of hemorrhage growth of factors related to secondary shearing (such as small vessel pathology) or time-dependent decay (such as hemostatic agents).

## Introduction

Spontaneous bleeding into the brain (intracerebral hemorrhage, ICH) represents a common and highly lethal form of stroke, with 3-month mortality of approximately 25% [1]. Large hemorrhage volume at presentation and subsequent hemorrhage expansion are strong predictors of poor post-ICH outcome [2], [3]. ICH in patients taking anticoagulant medications is associated with larger initial hemorrhage volumes than ICH in untreated individuals, greater likelihood of hematoma expansion, and an even higher 3-month mortality of 50% or greater [1], [4]–[7].

A major barrier to developing effective therapies to reduce the clinical burden of ICH is our poor understanding of the determinants of hemorrhage size and mechanisms for growth. Brain hemorrhages can occur as large symptomatic macrobleeds or small, clinically asymptomatic cerebral microbleeds [8]. An MRI-based analysis of patients with cerebral amyloid angiopathy-related hemorrhage [9] suggested macrobleeds and microbleeds do not form a single continuum of volumes, but instead comprise two distinct peaks that can be separated by a diameter cut-point of approximately 0.6 cm. The basis for this bimodal distribution has not been identified.

One possible mechanism that could generate distinct populations of microbleeds and macrobleeds is a threshold model, whereby once a nascent hemorrhage reaches a particular size, it continues to grow, becoming a macrobleed. Such a feed-forward mechanism for hemorrhage growth was suggested by early neuropathological studies showing secondary shearing of blood vessels surrounding a hematoma [10]. Fisher interpreted these data as hemorrhages growing in “domino” or “avalanche” fashion, one ruptured vessel triggering bleeding from the next.

We created a computer-based simulation to explore the properties of hemorrhages generated by secondary shearing of adjacent vessels. We analyzed the lesions’ size distribution, growth pattern, and response to prolonged decay of secondary shearing (to simulate the effects of anticoagulation).

## Methods

### Simulation of Hemorrhage Expansion

**Figure 1:**
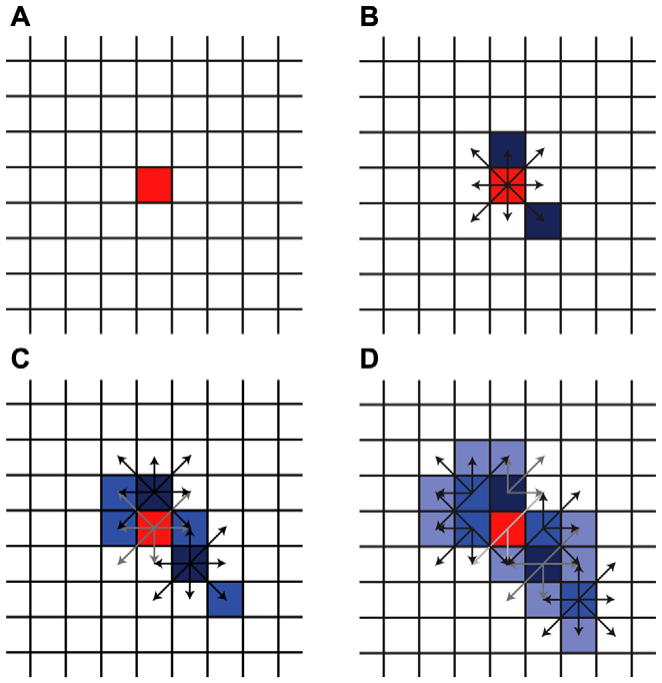
Simulated hemorrhage growth. Shearing forces from a primary rupture (panel A, red) generate possible secondary ruptures at neighboring segments (B, black arrows). Secondary vessel ruptures (dark blue) then contribute to possible further ruptures in subsequent cycles (C, D). The probability of adjacent rupture declines exponentially with each cycle, simulating coagulation (lightening arrows).

We simulated hemorrhage growth on a three-dimensional lattice comprised of virtual vessel segments, arrayed in a 150×150×150 cubic pattern. Each run of the simulation begins with an initial rupture of a vessel segment (the primary bleeding event) at the center of the lattice (Fig. 1A, shown as a two-dimensional lattice for simplicity). In the next cycle, the primary bleed introduces the possibility of rupture at each neighboring segment (Fig. 1B), simulating the process of secondary shearing [10]. Any secondary bleeding that indeed occurs contributes in turn to the possibility that other neighboring segments will rupture in subsequent cycles (Fig. 1C–D). The effect of multiple neighboring bleeds is additive, leading to possible cascading patterns of hemorrhage growth. The probability that any particular vessel will bleed is re-evaluated in each cycle.

To simulate the simultaneously occurring process of coagulation and clotting, the probability of secondary rupture contributed from a bleed to its neighbors decreases exponentially towards zero with each passing cycle. We also simulated the effect of increasing local pressure from surrounding tissue as a size-dependent decrease in the probability of further vessel ruptures. Hemorrhages were visualized using the geometry module of ROOT (http://root.cern.ch/).

### Simulation Parameters

To perform the simulation, three parameters of the model are specified. These are 1) an expansion constant *α* (unitless), representing the factor determining the likelihood of secondary shearing of adjacent vessels, 2) a time constant *τ* (units of cycles), representing the exponential falloff of the probability of shearing with increasing time since the onset of a particular vessel’s bleeding (simulating coagulation), and 3) a pressure constant β (units of ruptured vessels), representing the falloff in probability of further vessel rupture with increasing total hemorrhage volume (simulating local pressure effects). These parameters were systematically varied (see below) to determine their effects on the resulting simulated hemorrhages.

With these parameters specified, the total score for vessel segment *j* to rupture (*p_j_*) in a given cycle is given by the following expression, summed over all ruptured neighboring segments *i*:
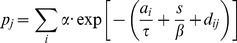
In addition to the specified parameters, the other variables in this expression are *a_i_*, the number of simulation cycles since neighbor vessel *i* ruptured; *s*, the total volume (measured in ruptured vessels) of the hemorrhage; and *d_ij_*, the center-to-center distance between vessels *i* and *j*. Whether a neighboring vessel segment ruptures in a given cycle is determined by whether the score exceeds a number between 0 and 1 randomly chosen for that segment and cycle. Neighbor vessels are those within two vessels in all directions (including corners).

### Analysis of Simulated Hemorrhagic Lesions

**Figure 2:**
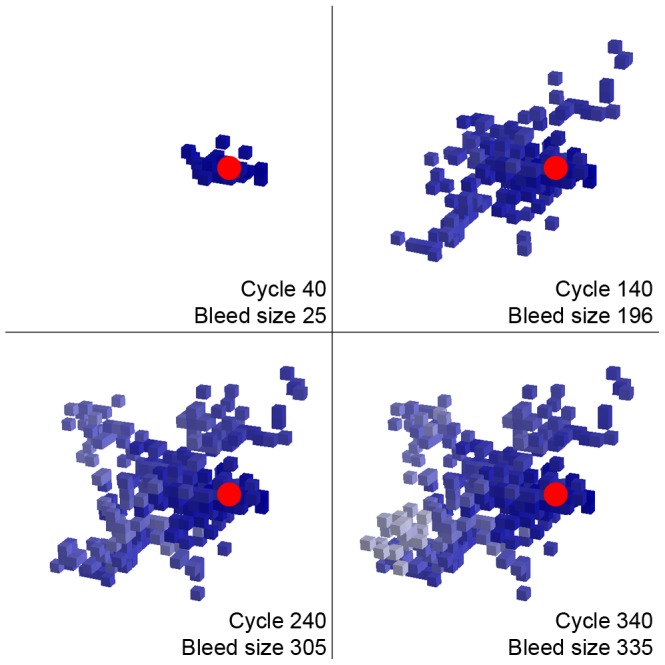
Representative expanding macrobleed. The originating vessel segment is shown in red, more recent ruptures in lightening shades of blue. No further growth occurred beyond cycle 340. Run parameters: *α* = 0.01, *τ* = 11, β = 500.

Each simulation was allowed to continue until hemorrhage growth ceased (see representative example in Fig. 2), defined as occurring when the highest score for a new vessel rupture at the site of the most recent rupture was 10^−6^. Following each run, the volume of the hemorrhagic lesion (measured as the total number of ruptured vessels) was recorded and the results binned using a natural log scale. Under certain parameter choices, we observed two peaks in the lesion size histogram, which we divided at the intervening bin with the lowest lesion count and designated as the microbleed (lower volume) and macrobleed (higher volume) peaks. Single vessel ruptures generally dominated the microbleed peak, whereas the macrobleed peak consisted of lesions resulting from secondary rupture.

For each choice of the parameters *α*, *τ*, and β, we ran 10,000 simulations and recorded the size distribution histograms. The simulations were performed on the University of California San Francisco QB3 Shared Computing Facility, a mixed-architecture Linux HPC grid with 3000 processor nodes. The 10,000 simulations typically required 100 to 3000 CPU-seconds, with larger hemorrhages requiring more simulation time.

### Parameter Search

We tested a range of values of the parameters *α*, *τ*, and β to look for regions in the parameter space that would yield a bimodal distribution of hemorrhage volumes as observed in patients. Each parameter was systematically increased through a range of values, holding the other two parameters constant. Increases in each parameter tend to enhance hemorrhage growth: *α* raises the likelihood of secondary ruptures, *τ* increases the time during which vessels continue to induce ruptures of their neighbors, and β enlarges the volumes at which local pressure prevents further ruptures. To improve the efficiency of the computations, we therefore followed a strategy of discontinuing further increases in a parameter when the generated population of macrobleeds was 8 times the population of microbleeds, as further increases would yield only further macrobleed overgrowth. The ranges tested were: *α*, 0.0001 to 0.05 in steps of 0.0001; *τ*, 0.1 to 50 in steps of 0.1; and β, at 500 and 5000.

**Figure 3:**
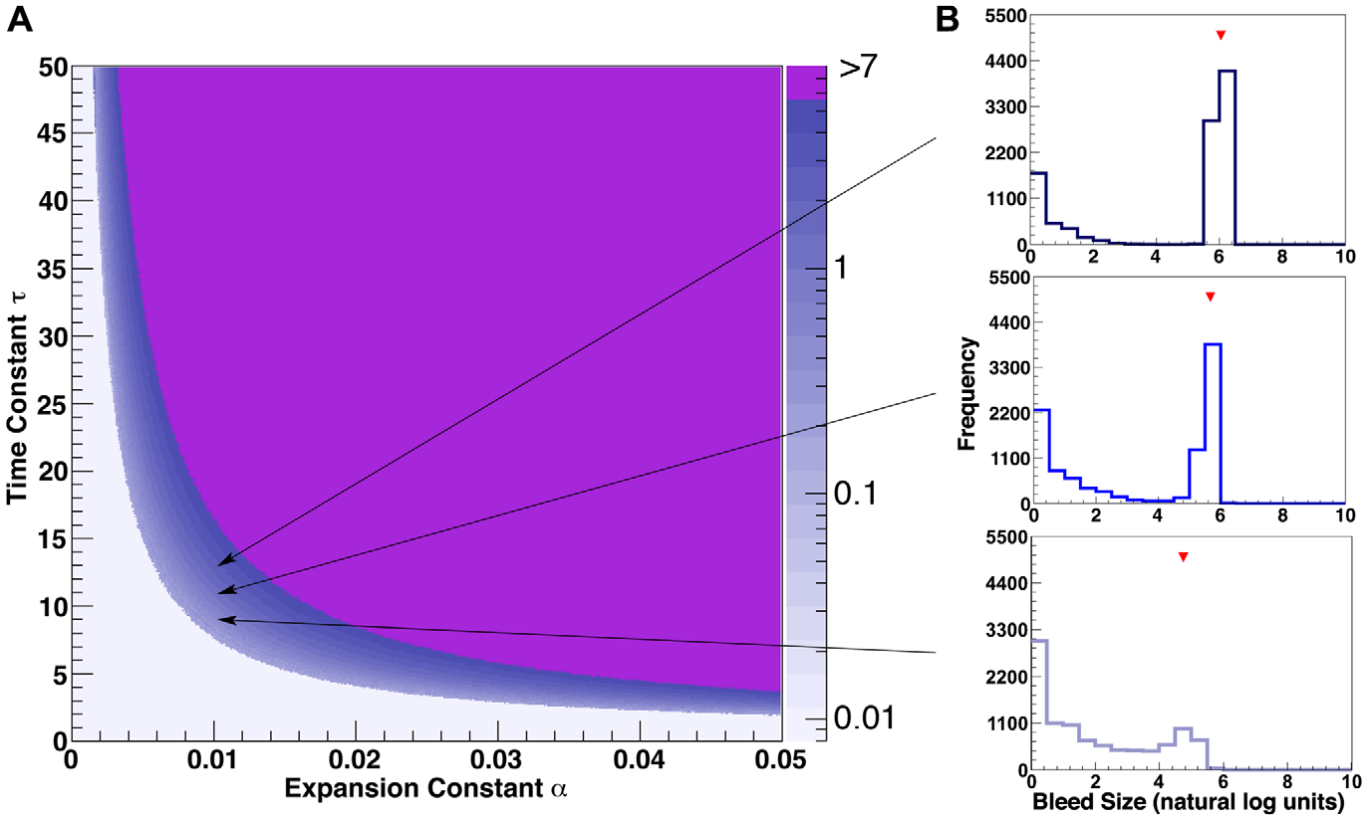
Effect of model parameters on distribution of hemorrhage volumes. Panel A depicts combinations of expansion coefficient *α* and coagulation time constant *τ (*β = 500). Z-scale is # macrobleed/# microbleeds (log 10 ratio). Panel B shows sample histograms for bimodal conditions with three different levels of decay constants (top: *τ* = 13; middle: *τ* = 11, bottom: *τ* = 9, all with *α* = 0.01). Red marks in panel B indicate median size of macrobleeds at those parameters (log value 4.71 for *τ* = 9, 5.62 for *τ* = 11, and 6.02 for *τ = *13, corresponding to the values for macrobleed volume shown in Table 1).

To simulate alterations in blood coagulation (as occur in patients taking anticoagulant medications), we also performed more in-depth analyses on three values of the “coagulation factor” *τ* (9, 11, and 13) at fixed α (0.01) and β (500) values (Table 1). The three values of *τ* were selected to represent a physiologically realistic range of the coagulation process in which both microbleeds and macrobleeds could occur (illustrated in Fig. 3B). For these investigations, the full growth histories of 10,000 simulated lesions were stored and analyzed. In addition to determining lesion volumes and macrobleed:microbleed ratios from the resulting histograms as above, we used the growth histories to generate values for the rate and duration of expansion. Rate of expansion was evaluated by fitting a sigmoid growth curve to hemorrhage size as a function of time (size = *A*/(1+*e* ^−*B*(*t*−*C*)^) where the maximal growth rate is given by AB/4 and the parameters A, B, and C selected by minimizing the chi-square fit with the observed data using the Minuit minimization package implemented in ROOT). Duration of expansion was the number of cycles until growth ceased as defined above. We also performed additional simulations in which an initial “anticoagulated” value of *τ* = 13 is abruptly replaced during the course of hemorrhage expansion with the “normal coagulation” value of *τ* = 9.

**Table 1:** Analysis of macrobleeds under various simulated anticoagulant conditions.

Decayconstant	Proportion ofmacrobleeds	Macrobleed volume	Total duration of macrobleed growth	Maximal rate of macrobleed growth
*τ*	(% total hemorrhage events)	(median vessels, interquartile range)	(median cycles, interquartile range)	(median cycles^−1^, interquartile range)
9	26.9	111 (70–153)	198 (153–247)	0.95 (0.65–1.32)
11	53.2 *	276 (238–310) *	293 (250–347) *	2.04 (1.53–2.56) *
13	70.0 *	412 (382–440) *	310 (271–357) *	3.46 (2.92–4.03) *

All data were derived from 10,000 hemorrhage simulations (see Methods). P-values (in comparison to next lower value of *τ*) were determined by chi-square (proportion of macrobleeds) or Mann-Whitney test (volume, duration, rate).

* p<0.001.

Statistical comparisons of continuous parameters (growth rate, duration, and maximum hemorrhage volume) were evaluated using Mann-Whitney tests because of non-normal distributions. Comparisons of the fraction of microbleeds vs. macrobleeds at different parameters were performed using a chi-square test. The independent contributions of growth rate and duration to final volume were analyzed by partial correlation in a model that also included a categorical variable for the three examined values of *τ*.

## Results

Depending on model parameters, a subset of simulated primary vessel ruptures grew into larger hemorrhages. Representative expanding macrobleeds are shown at discrete time points (Fig. 2) and as a movie (Movie S1). Expansion of primary vessel ruptures into larger hemorrhages tended to occur asymmetrically in apparently arbitrary directions rather than concentrically around the site of origin. Smaller lesions (single vessel ruptures or small clusters) generally ceased growing solely by failure to trigger further ruptures, thus “self-terminating” without the additional volume-dependent contribution to reduced rupture probability (i.e., even with ß set to high values). For larger lesions, termination occurred by the volume-dependent pressure effect, such that at large values of ß these lesions continued growing to the simulated lattice limits.

Under conditions of expansion, hemorrhage volume histograms yielded distinct peaks of smaller (microbleed) and larger (macrobleed) volume lesions. Whether microbleeds or macrobleeds predominated varied according to parameter choice for expansion (*α*) and decay (*τ*) of secondary rupture. The dark blue central band in Figure 3A represents combinations of *α* and *τ* that generate a macrobleed:microbleed ratio in a similar range to the clinically observed bimodal distribution in ICH patients [9]. Variations in ß typically did not affect the macrobleed:microbleed ratio, but rather only the median volume of the lesions comprising the macrobleed peak (data not shown).

To further understand the effects of varying the decay time constant (corresponding to the physiologic property of coagulation speed), we performed more in-depth analyses at three values of *τ* (Table 1). These values of *τ* were selected to simulate the effects of prolonged coagulation within a narrow, physiologically realistic range of macrobleed:microbleed ratios. As shown in Table 1 and Fig. 3B, increasing values of *τ* yielded 1) higher macrobleed:microbleed ratios (i.e. a higher likelihood that a given vessel rupture would expand into the macrobleed range), and 2) larger median volumes for the macrobleeds. To explore the mechanism underlying the larger macrobleed volumes achieved at higher values of *τ*, we further analyzed the duration and rate of hemorrhage expansion. We found that both the growth duration (number of cycles until ceasing growth) and the maximal growth rate (the rate parameter in the fitted sigmoid function) increased with higher *τ*, along with an increase in hemorrhage size (Table 1). Controlling for the different values of *τ*, maximal growth rate and duration each contributed independently to final hemorrhage volume (partial correlation coefficients 0.88 and 0.82 respectively, p<0.001 for each).

**Figure 4:**
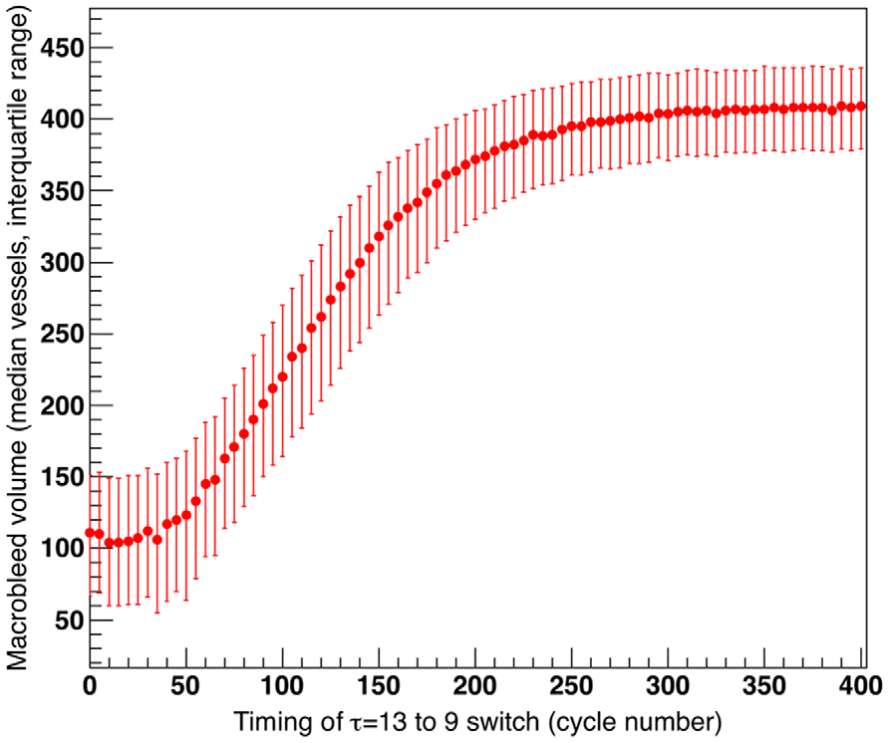
Effect of simulated reversal of anticoagulation. The simulated anticoagulation condition (*τ* = 13) was abruptly switched to simulated normal coagulation (*τ* = 9) at the time points indicated on the x-axis during the course of hemorrhage expansion. Simulations were otherwise performed as described in Table 1.

To simulate the situation where anticoagulation is therapeutically reversed after hemorrhage is detected, we performed additional analyses of the effect of abruptly switching from the simulated anticoagulation condition (*τ* = 13) to simulated normal coagulation (*τ* = 9) at various time points during the course of hemorrhage expansion. Even under these unrealistically rapid conditions of reversal (a process that can require 5 hours or more in clinical practice [11]), only very early switches were effective in substantially limiting final hematoma size (Fig. 4). Reversals performed at time points close to the median duration of macrobleed growth under normal conditions (198 cycles; Table 1) prevent very little of the anticoagulation-related enlargement in median hemorrhage size (Fig. 4).

## Discussion

We report that a simulation based on rules directly derived from neuropathological observations in hemorrhagic brain tissue [10] can give rise to a range of features similar to those of clinically observed ICH. Among these are 1) a bimodal distribution of microbleeds and macrobleeds (as found in a study of 46 patients with lobar ICH [9]), 2) an asymmetric rather than concentric pattern of hemorrhage expansion [12], 3) increased incidence of macrobleeds under conditions simulating prolonged coagulation (as observed in patients treated with anticoagulant medication [13], [14]), and 4) larger macrobleed volumes under the anticoagulated condition (also observed clinically [5]–[7]). The similarities between simulated and observed results are consistent with the possibility that the current model captures at least some of the key mechanistic features of clinical ICH expansion.

The report by Fisher [10] motivating the current model noted at least 24 sites of recent arterial rupture within a single pontine hemorrhage, mostly small and at the hematoma periphery. Based on their position, absence of evident chronic arterial disease, and large number, Fisher surmised that “all or most of the [vessel ruptures] were the secondary result of a mechanical disruption caused by blood escaping from a primary site of hemorrhage,” creating an “avalanche” of bleeding vessels. We previously suggested secondary shearing could generate a bimodal distribution of ICH volumes [9]; the current simulation supports this idea.

To simulate the effects of therapeutic anticoagulation and reversal, we further investigated the effects on hemorrhage growth of varying the decay time constant. Small increases in *τ* (all within the range of parameters that yielded both microbleeds and macrobleeds, Fig. 3) resulted in both faster rates and longer durations of hemorrhage growth (Table 1), each of which contributed to larger final macrobleed volumes. Growth rate and duration have not been extensively studied in clinical ICH, but there is evidence that prolonged duration may indeed be a feature of anticoagulant-related hemorrhage [4]. Finally, our model suggests that the effects of anticoagulation may determine final hemorrhage size relatively early in the course of hematoma growth (Fig. 4), consistent with the difficulties encountered by several groups in demonstrating beneficial effects of anticoagulation reversal [15]–[19].

Our computer-based model is intended as a tool for generating and testing hypotheses on the factors that affect hemorrhage growth. While we attempted to model some biological properties of *in vivo* hemorrhages, we excluded several aspects of bleeding for simplicity. Lesion volume, for example, was calculated as number of ruptured vessels, neglecting the likely vessel-to-vessel variations in volume of extruded blood. The model also does not account for the location, heterogeneity, and anisotropy of the surrounding brain tissue, which likely contributes to the direction of hemorrhage expansion in patients. It is interesting in this context that the simulation nonetheless generated a considerable degree of asymmetric growth, suggesting that the secondary shearing and clotting processes may also contribute to this feature. We further note the lack of a straightforward or quantitative connection between model parameters (measured in arbitrary units of simulation cycles and virtual vessel segments) and physical parameters of human hemorrhage. Addressing these limitations will likely require further studies correlating simulated results to observed clinical data and greater model complexity.

A potential application for future iterations of this model will be to dissect the individual effects of factors related to secondary vessel shearing (*α* in our model) and time-dependent decay (*τ*) on particular aspects of hemorrhage growth. Although they are parameters in a virtual model, both *α* and *τ* may correspond to identifiable physical features in ICH. Likelihood of secondary vessel rupture, for example, would be expected to relate to small vessel structure. Alterations of vessel structure have indeed been postulated to account for some findings in hemorrhage expansion, such as the effect of the apolipoprotein E ε2 allele on growth of cerebral amyloid angiopathy-related ICH [20], [21]. Time-dependent decay of rupture, conversely, would be expected to depend on hemostatic factors such as the coagulation cascade, another observed determinant of hemorrhage volume [22], [23]. Future comparisons of observed to simulated data might further help unravel the mechanisms of other interventions affecting hemorrhage expansion, such as acute blood pressure lowering [24], [25]. As final hematoma size is a powerful determinant of patient outcome [2] and continued expansion after clinical presentation occurs in approximately one-quarter to one-third of macrobleeds imaged early after presentation [24]–[26], the identified steps in this process will be important future targets for exploration and treatment.

## Supporting Information

Movie S1 Examples of simulated hemorrhages. All simulated lesions were allowed to grow until termination. Run parameters: *α* = 0.01, *τ* = 10.0, β = 500 (within the region of bimodal distribution in Figure 3A). See legend to Figure 2 for color scheme.
(MOV)
